# Investigating the Mechanism behind ‘Ant Nest’ Corrosion on Copper Tube

**DOI:** 10.3390/ma11040533

**Published:** 2018-03-30

**Authors:** Riky Stepanus Situmorang, Hideki Kawai

**Affiliations:** 1 Division of Engineering, Muroran Institute of Technology, 27-1, Mizumoto-cho, Muroran, Hokkaido 050-8585, Japan; 2 Database Researcher of Department of Mechanical System Engineering, Muroran Institute of Technology, 27-1, Mizumoto-cho, Muroran, Hokkaido 050-8585, Japan; hdkawai0@mmm.muroran-it.ac.jp

**Keywords:** copper tube, “ant nest” corrosion (ANC), pitting attack, comproportionation reaction, disproportionation reaction

## Abstract

A research investigation of “ant nest” corrosion (ANC) on copper tube was conducted in terms of the variables of the corrosion potential and pH value in 10^3^ ppm copper formate solution over 20 days. The paper presents the surface and cross-sectional observations and examines Cu_2_O and H_2_O as the stable chemical species produced. A Cannizzaro reaction as a disproportionation reaction from formic acid and a comproportionation reaction from the metallic copper tube and copper formate solution critically influenced the ANC mechanism. The paper also categorizes the ANC attack as a rapid reaction system from the electrochemical point of view by using a polarization resistance curve.

## 1. Introduction

Global warming contributes dramatically to an increasing number of applications of air conditioners and refrigerators [1]. Due to the many advantages of copper tube, especially its thermal conductivity, copper is commonly used in an air conditioner and refrigerator in a household or public building [2,3]. Therefore, many researchers in the company and public research realms have been studying the problems of copper tubes such as corrosion leakage. Ant nest corrosion (ANC) on the copper tube is one of the most severe problems occuring in air conditioners these days [4,5,6,7,8]. However, the ANC issue is important to solve to maintain the efficiency of the air conditioner. This issue also can contribute to the energy use that can influence global warming [9].

The ANC or formicary corrosion has corroded pitting hole traces of several micron size morphologies that are similar to the nest of ants. This typical corrosion was firstly reported by Edwards et al. in the 1970s [10]. Characteristics of the ANC are: (1) the ANC can not be observed by bare eyes’ inspection because its size is about several microns; (2) forms of its corrosion are complicated; it looks like a tunnel and has a number of wormholes randomly in the thickness direction of the copper tube; (3) the corrosion rate might be fast; for example, the corrosion rate of the phosphorus deoxidized copper tube was shown as approximately 10 μm/day (about 0.3 mA cm^−2^) [11].

Carboxylic acids such as formic and acetic acid are the agent behind the mechanism of ANC [12,13]. However, the form of corrosion, branching pits, and wormholes attack are depended on the compound agent in the environment. The formic acid atmosphere provokes ANC on the copper tube to be a more perplexing branch hole rather than the acetic acid atmosphere [14,15,16]. In vapor test conditions, acetic acid has a higher corrosion rate than formic acid in copper tube [17]. However, in some conditions, ANC phenomena have a closeness to stress corrosion cracking and stress cracking [7,18,19,20].

There are two kinds of copper tube commonly used in daily life; a phosphorus deoxidized copper tube (Cu-PDC) and an oxygen-free copper tube (Cu-OFC) without phosphorus. Sakai et al. [21] conducted research on the ANC of both copper tubes in acetic acid solution, a formic acid solution. This found that phosphorus in copper is not a crucial factor for the generation of ANC. In addition, there are many research works on the prevention of ANC in copper tubes such as the development of surface treatments on copper tube [22,23]. However, ANC still occurs in copper that has had surface treatment. The surface treatment only postpones the ANC attacking the copper tube.

The corrosion mechanism of ANC in copper has been reported by many researchers [4,5,6,7,8,16,24] but the phenomena of ANC which is complicated makes necessary an explanation about the corrosion mechanism in more complex solutions, except acetic acid or formic acid. O. Seri et al. [25] explain that in a neutral or alkali environment the reaction between copper and formic acid can produce copper formate. The proportionate reactions may be involved between metallic copper with copper formate ions. Moreover, this can be used as a considering factor to explain the mechanism.

In order to understand the underlying behavior of the ANC, this research is focused on the corrosive environment of copper formate solution. The reasons why copper formate solution is employed as a test solution are: (1) copper reacts with formic acid to form copper formate as a corrosion product; therefore, the pitting cavities will be occupied with the copper formate solution; (2) the replacement of the pit solution to the bulk solution is complicated due to the narrow pitting mouth of the ANC; therefore, the copper formate solution probably resides in the pitting cavities; and (3) the comproportionation reaction between a copper formate and metallic copper is indispensable for the explanation of the ANC that will be discussed later.

## 2. Materials and Methods

### 2.1. Test Material

The sample specimen was commercial phosphorus deoxidized copper tube (JIS1220, UACJ Cooperation, Tokyo, Japan, ∅15 mm × 0.7 mm, Cu ≥ 99.90%, P: 0.015~0.040%). All samples were rinsed with acetone as a pretreatment and then washed with deionized water. All the specimens were air-dried before the test.

### 2.2. Test Solution

Test solutions were copper formate (98 wt % Cu(COOH)_2_, Wako Pure Chemical Ltd., Tokyo, Japan) as a solvent of the ion-exchanged water. The test solution was adjusted to 10^3^ ppm Cu(COOH)_2_ concentration. The solution temperature was room temperature (about 298 K). In the initial condition, the solution pH was 5.5, electric conductivity κ was 86 mS/m, and the dissolved oxygen (DO) was around 5 ppm. For altering the pH 5.5 of solution to be pH 3, reagent grade 98 wt % formic acid (HCOOH, Wako Pure Chemical Ltd., Tokyo, Japan) was added.

### 2.3. Observation Method

Copper tube specimen was immersed in 10^3^ ppm Cu(COOH)_2_ solutions, respectively, of pH 5.5 and 3 (added HCOOH). The sample immersed in the test solutions was periodically observed at 0 day, 5 days, 10 days, 15 days, 20 days and 30 days after cleaning by ion-exchanged water and air-dried. The surface observation was carried out using a Microscope Lasertec Optelics Hybrid (Lasertec L3SMZ, Lasertec cooperation, Tokyo, Japan). In a cross-section observation, a part of the sample was cut, embedded in the epoxy resin, then polished with the fine polishing machine (Polis IMT-P2, IMT Cooperation, Tokyo, Japan) and finally using microscope BX51M-33MB (Olympus cooperation, Tokyo, Japan). The cross-section observation was carried out whether the ANC occurs or not. The areas of copper tube attacked by ANC were investigated then with an electron probe micro-analyzer (EPMA) (JEOL JXA8900R, JEOL Ltd., Tokyo, Japan).

### 2.4. Measurement Method

An electrochemical measurement system (HZ7000 Hokuto Denko Ltd., Tokyo, Japan) was employed for polarization measurements. The scan rate was 0.1 mV/s. The surface of the copper specimen was masked with insulating tape and silicon resin, except for the exposed surface area of 5.6 cm^2^. As a reference electrode, an Ag/AgCl electrode (DKK-TOA Co., Tokyo, Japan) in the saturated potassium chloride solution was used. In this paper, the electrode potential related to the Ag/AgCl reference electrode (V vs. SSE) was simplified as V unless otherwise noted.

## 3. Results

### 3.1. Specimen Observation

**Figure 1:**
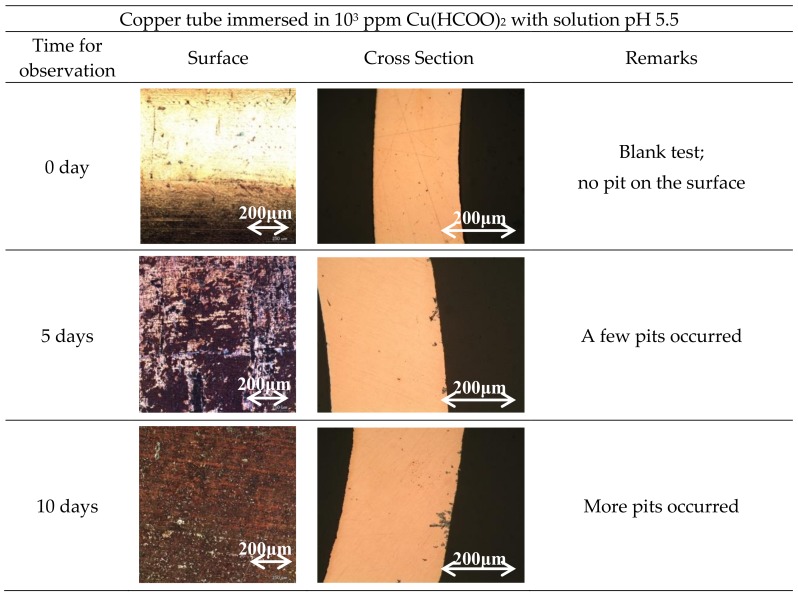

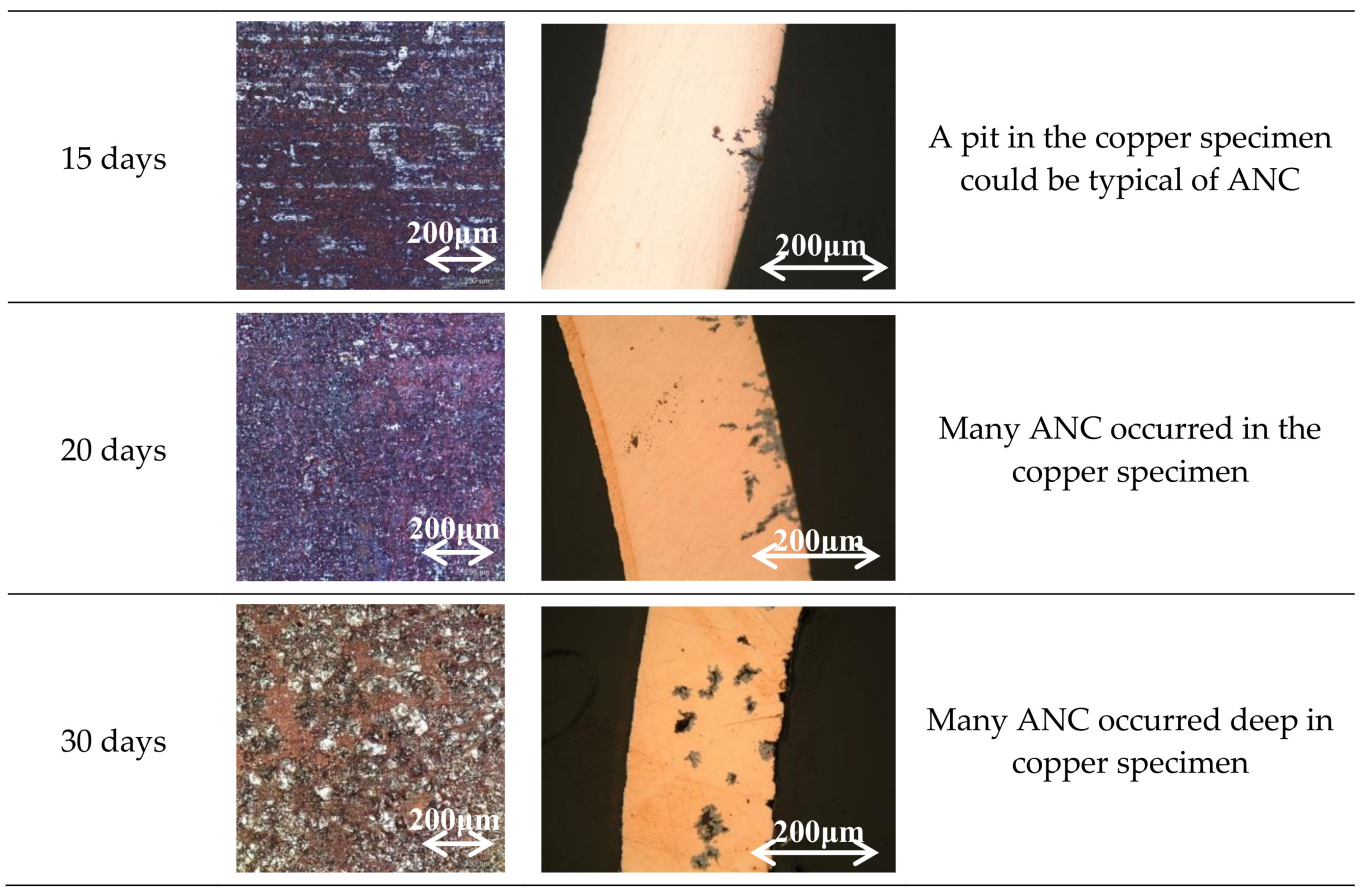
Surface and cross-section observations of Cu tube specimen immersed in 10^3^ ppm Cu(HCOO)_2_.

**Figure 2:**
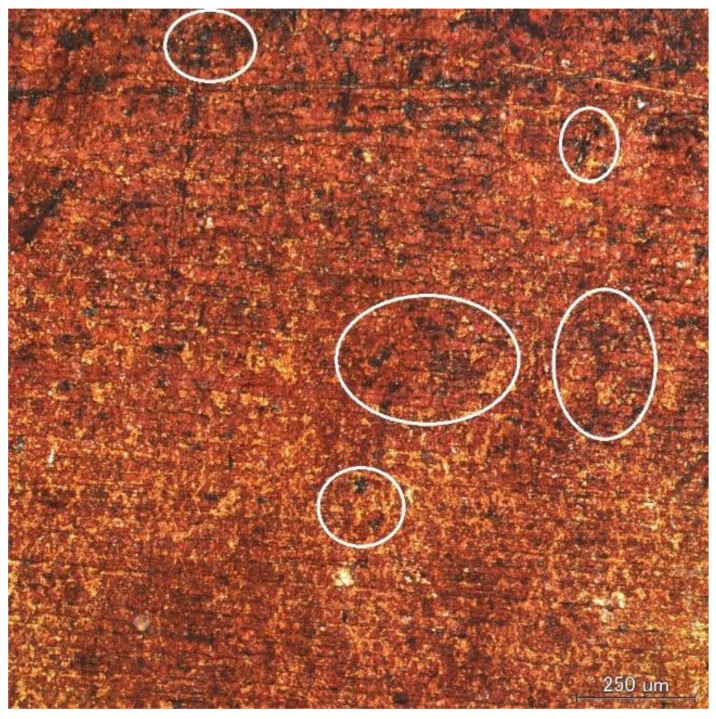
Surface observations of Cu tube specimen immersed in 10^3^ ppm Cu(HCOO)_2_ after 10 days immersion.

Figure 1 shows the results of surface and cross-section observation of copper tube in 10^3^ ppm Cu(COOH)_2_ solution with periodic time observation. The surface colors of the copper tube in the visual observation were reddish brown, reddish purple, or black as presented in the optical microscope of the left table of Figure 1. It took 10 days until all of the surfaces were covered by corrosion. The small stain black holes were found on the copper tube surface after being immersed for 10 days as shown in the white circle in Figure 2. It was assumed as the initial stage of the pitting attack of ANC which was confirmed by the cross-section observation. The embryo of ANC started after 10 days’ immersion, and the ANC was obviously observed on the copper tube after being immersed for 20 days. Therefore, the measurement of natural corrosion potential and the polarization curve were done after 20 days. Increasing immersion time increased the number of ANC on the copper tube.

**Figure 3:**
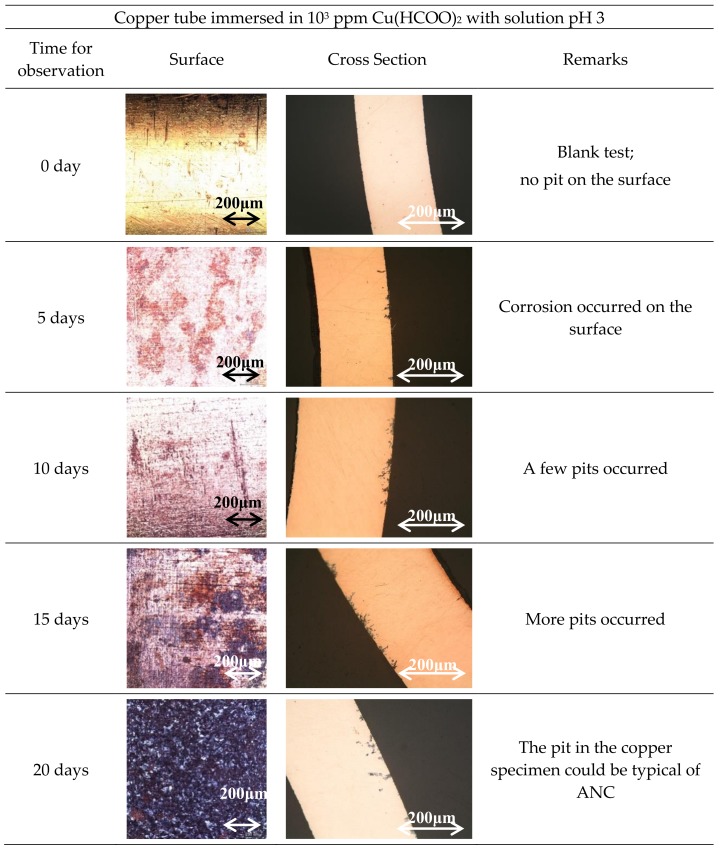

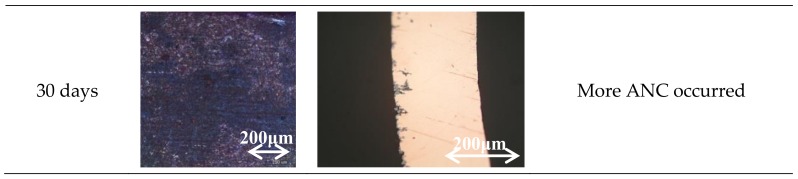
Surface and cross-section observations of Cu tube specimen immersed in 10^3^ ppm Cu(HCOO)_2_ with solution pH 3.

In order to study the influence of pH on the copper tube in 10^3^ ppm Cu(COOH)_2_ solution, the copper tube was immersed in 10^3^ ppm Cu(COOH)_2_ solution that was adjusted to pH = 3 using HCOOH. Figure 3 shows the surface and cross-section observation of the copper tube in 10^3^ ppm Cu(COOH)_2_ solution adjusted to pH = 3 with periodical observations. Compared to pH = 5.5; the corrosion occurred more prominently on the surface of the copper tube in this condition. The surface of the copper was covered by the corrosion after 20 days immersion and spread on the surface rather than a pitting attack into the copper tube specimen, as confirmed by the cross-section observation. The embryo of the ANC started after 20 days’ immersion, and the ANC was obviously observed on the copper tube after being immersed for 30 days. 

**Figure 4:**
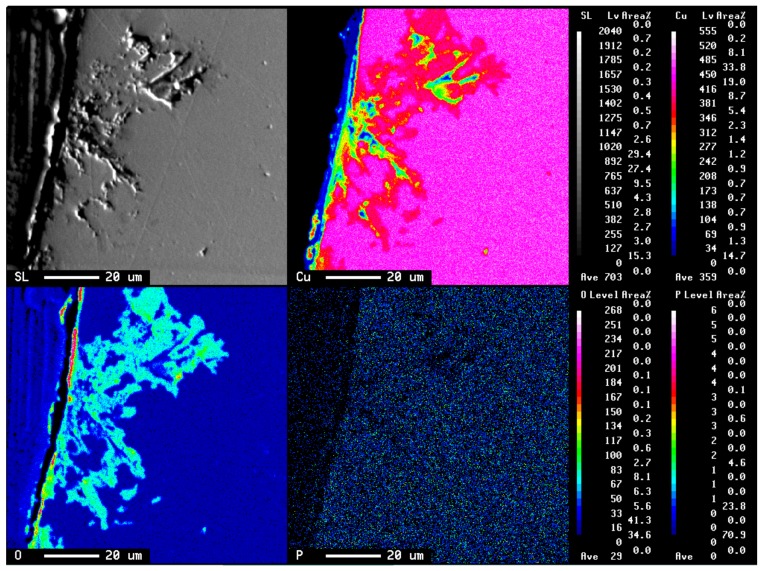
The result of scanning electron microscope (SEM) image of the copper tube after being immersed 15 days in 10^3^ ppm Cu(HCOO)_2_; electron probe micro-analysis (EPMA) led to corresponding elements of copper, oxygen, and phosphorus.

**Figure 5:**
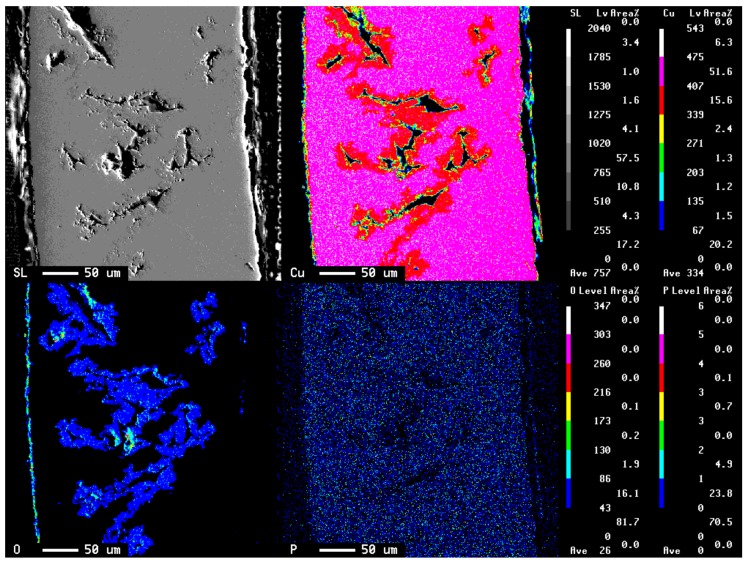
The result of SEM image of the copper tube after being immersed 30 days in 10^3^ ppm Cu(HCOO)_2_; EPMA led to corresponding elements of copper, oxygen, and phosphorus.

Figure 4 shows the result of a scanning electron microscope (SEM) picture and EPMA analysis of the cross-section of the copper tube that was immersed in 10^3^ ppm Cu(COOH)_2_ solution with pH 5.5 after 15 days immersion time. This EPMA analysis was conducted to detect the presence of copper, oxygen, and phosphorus elements. Results show that the presence of phosphorus was at a low level in all areas. However, the presence of copper was almost in all areas and was higher in the border of the pitting hole, whereas the presence of oxygen was only detected in the pitting hole of the copper tube and the surface. This EPMA analysis result shows that copper and oxygen had an important role in the pitting attack. In addition, the EPMA analysis was also conducted on the copper tube after 30 days immersion time to detect the level differences of copper and oxygen elements on the copper tube as shown in Figure 5. As a result, It was found that oxygen was detected at a low level in the pitting hole after 30 days immersion time compared to 15 days. Therefore, after long immersion periods of copper tube it was concluded that the oxygen found it difficult to exist deep in the pitting hole.

### 3.2. E_corr_, pH-Time

**Figure 6:**
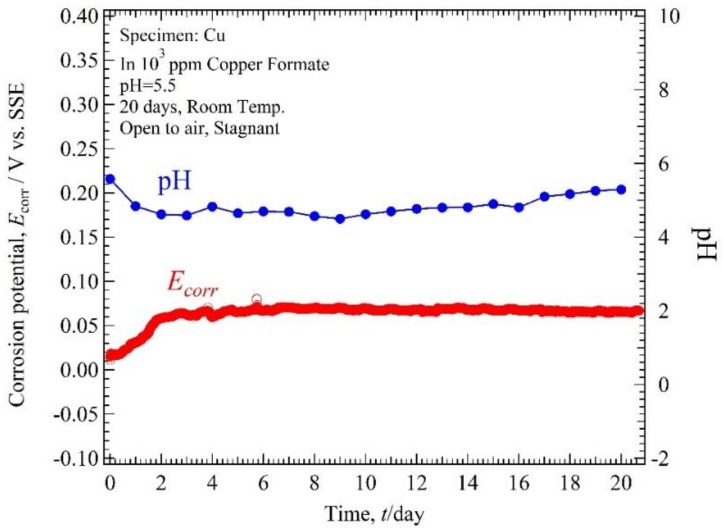
Corrosion potential, solution pH and time plot of Cu tube specimen in 10^3^ ppm Cu(HCOO)_2_ solution with solution pH 5.5.

**Figure 7:**
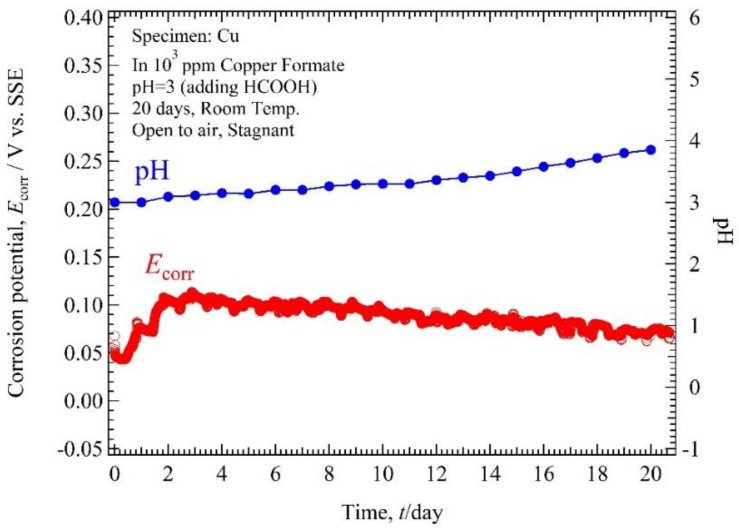
Corrosion potential, solution pH and time plot of Cu tube specimen in 10^3^ ppm Cu(HCOO)_2_ solution with solution pH 3.

The monitoring results of *E*_corr_ and pH of copper in 10^3^ ppm Cu(COOH)_2_ solution with pH 5.5 and 3 are shown in Figure 6 and Figure 7. *E*_corr_ for pH 5.5 was in the range 0.01 to 0.08 V vs. SSE for 20 days immersion time. *E*_corr_ started from 0.01 V vs. SSE and then increased rapidly to 0.06 V vs. SSE in 3 days and after that was almost constant in the range 0.06 to 0.08 V vs. SSE. The solution pH measurement shows that at first immersion time the solution pH was 5.58 and it decreased to 4.59 in 3 days, then after that it increased slowly until the end of the measurement time to 5.29. *E*_corr_ for pH solution 3 was in the range 0.04 to 0.11 V for 20 days immersion time. *E*_corr_ started from 0.04, then increased to 0.1 in 3 days immersion time, and decreased slowly to reach 0.07 V until the end of the measurement. The solution pH measurement shows that at the first immersion time the solution pH was 3 and continuously increased to 3.85 until the end of the measurement time.

### 3.3. Polarization Curve

**Figure 8:**
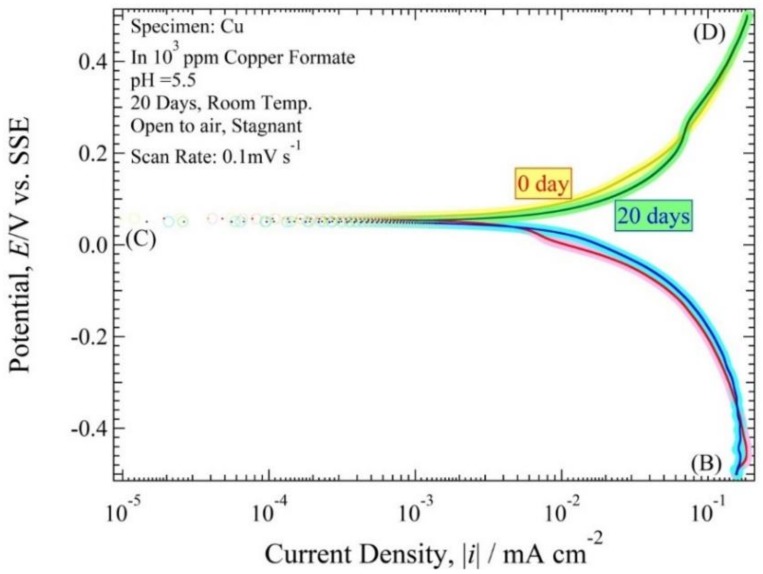
Polarization curve of Cu tube specimen in 10^3^ ppm Cu(HCOO)_2_ solution at the immersion period of 0 days and 20 days with solution pH 5.5.

**Figure 9:**
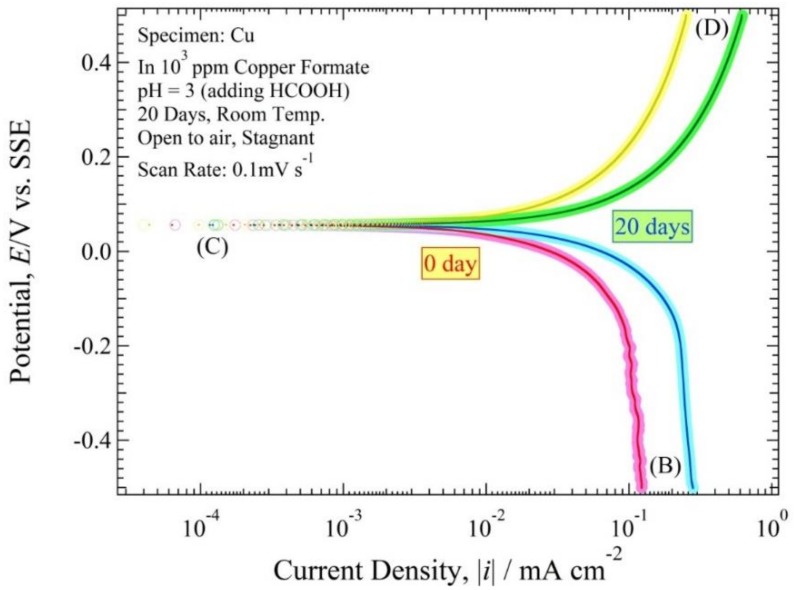
Polarization curve of Cu tube specimen in 10^3^ ppm Cu(HCOO)_2_ solution at the immersion period of 0 days and 20 days with solution pH 3.

The cross-sectional observation shows that the ANC obviously occurred on the copper tube after being immersed for 20 days. Therefore, it was shown in the polarization curve measurements observed at 0 days and 20 days. Figure 8 and Figure 9 show the polarization curve of copper tube in 10^3^ ppm Cu(COOH)_2_ solution with pH 5.5 and 3, respectively. After holding at open circuit potential (*E*_ocp_) for about 1 ks, the cathodic operation was performed from the *E*_ocp_ to point (B), and from point (B) the polarization operation was reserved to point (C). Then the anodic current begins to be observed at point (C), and beyond point (C) the anodic current exponentially flowed. At point (D) the polarization operation was finished. All of the polarization curve measurements show the current route of (B)–(C)–(D), because the first cathodic route of *E*_ocp_–(B) is almost same as the reserve route of (B)–(C). *E*(*i*) curves of 0 day immersion and 20 day immersion visually exhibited almost the same curve.

In every solution pH condition, the *E*(0) value for 0 days and 20 days in pH solution 5.5 were 0.04 and 0.05 V vs. SSE, and the |*i*| plot around (|*i*| < 0.16 mA cm^−2^) for 0 day and (|*i*| < 0.09 mA cm^−2^) for 20 days. However, the *E*(0) value for 0 days and 20 days in solution pH 3 were same, 0.056 V vs. SSE with the |*i*| plot around (|*i*| < 0.25 mA cm^−2^) for 0 days and (|*i*| < 0.62 mA cm^−2^) for 20 days. However, the trend of the line was almost identical after being immersed 20 days. However, it was better to use a cross-section observation to detect the ANC on the copper tube rather than a polarization curve observation.

### 3.4. Polarization Resistance Curve

**Figure 10:**
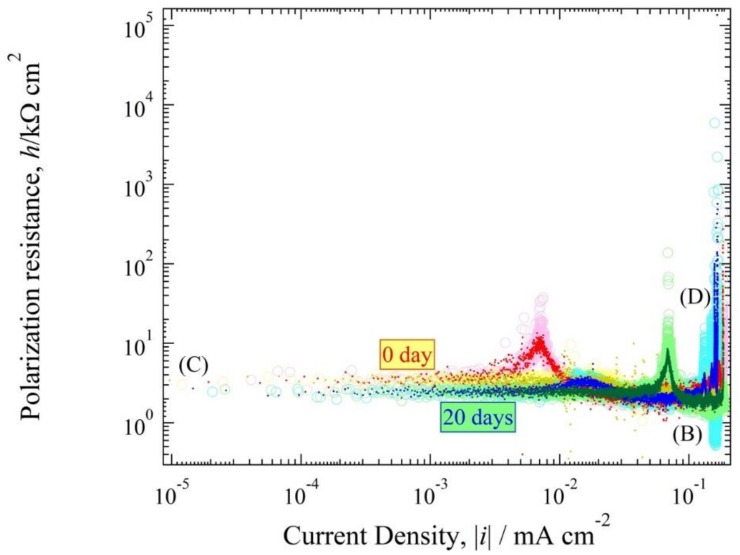
Polarization resistance curve of Figure 8.

**Figure 11:**
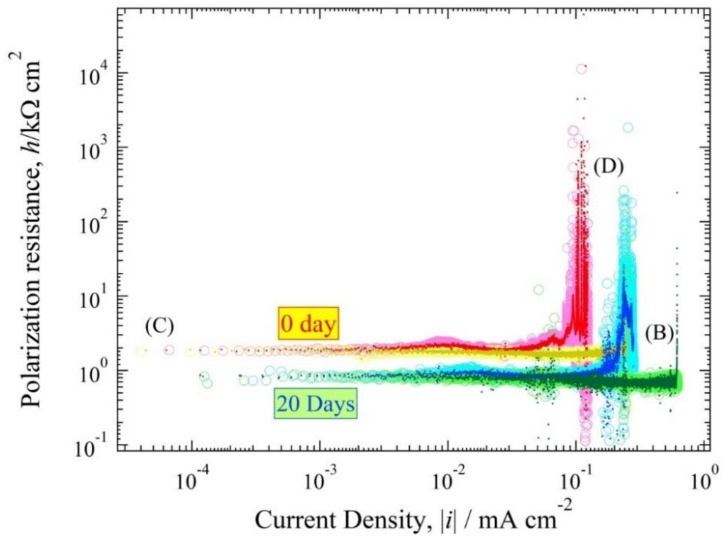
Polarization resistance curve of Figure 9.

The polarization resistance curve, *h*(*i*) (=d*E*/d*i*) often has the advantage of making a distinctive shape compared to the *E*(*i*) expression curve. Figure 8 and Figure 9 were converted to log*h*~log|*i*| expression, as show in Figure 10 and Figure 11. The *h*(0) values for 0 days and 20 days in pH solution 5.5 were 3 kΩ cm^2^ and 2.7 kΩ cm^2^. The *h*(0) values for 0 days and 20 days in pH solution 3 were 1.9 and 0.8 kΩ cm^2^. The *h*(0) exhibited an almost horizontal line with a strong characteristic resemblance, except for the copper tube that was immersed in pH solution 5.5; the peak confirmed on the cathode polarization resistance curve was of 7 × 10^−2^ mA cm^−2^ for 0 days immersion time and the anode polarization resistance curve of 6 × 10^−2^ mA cm^−2^ for 20 days immersion time. These typical horizontal lines in the polarization curves show that the reaction for the ANC was a rapid reaction system [6,13]. Also, the branch curve for anodic polarization resistance and cathodic polarization resistance were almost overlapped.

## 4. Discussion

### 4.1. Relation of E_corr_, pH Solution, and Immersion Time

The ANC occurred in the phosphorus deoxidized copper tube immersed in 10^3^ ppm Cu(COOH)_2_ solution, respectively, of pH 5.5 and 3 (added HCOOH). Figure 1 and Figure 3 show the unique profile of ANC both on the surface and in the pitting cross-section. The reddish brown (Figure 1 and Figure 3 (0 days to 10 days)), reddish purple (Figure 1 (15 and 20 days) and Figure 3 (20 days)), or black color (in 30 days in both condition) on the surface of copper tube confirm that cuprous oxide exists as the corrosion product that had been generated along the mechanism [2,26]. The existence of black color on the surface in the late immersion time is a sign of a step of the oxidized copper as reported by Eliot et al. [4]. The small stain black holes shown in Figure 2 confirm that the ANC started from a very localized attack [5] which is difficult to observe with bare eyes.

By comparing Figure 1 and Figure 3, it can be seen that the decreasing pH solution prevents the rising amount of ANC on the copper tube. In addition, the solution pH influenced the prominent pitting attack condition of the copper tube. Figure 1 shows that the corrosion that occurred in solution pH 5.5 had the same prominent rate on the surface and deep in the pitting attack. Figure 3 shows that the corrosion had a more prominent influence on the surface than in the pitting attack in solution pH 3. But in both conditions of the late immersion time, the branch of pitting corrosion was random with complex structure tunnels, which means all of the corrosion is typical of the ANC [8,15,16,27]. The existence of a single chemical compound (Cu(HCOO)_2_) in the solution in Figure 1 may be a strong influencing factor. But in Figure 3, this might start from the dissociation reaction between HCOOH and Cu(HCOO)_2_ and after the solution became stagnant, which means Cu(HCOO)_2_ existed more than HCOOH in the solution, then the corrosion increased and attacked deep in the copper tube.

**Figure 12:**
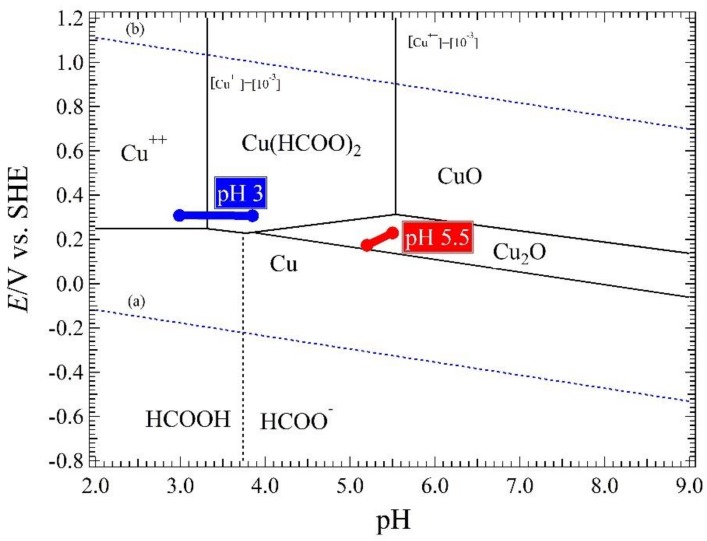
*E*-pH Diagram of Cu and Cu(HCOO)_2_ with pH condition 5.5, with the assumption that the concentration of total dissolved copper ions [Cu^++^] was 10^−3^ mol kg^−1^.

The *E*-pH diagram of Cu with Cu(HCOO)_2_ in every pH condition is shown in Figure 12 [16,28]. Chemical potential values for the calculation are listed in Table 1 [29]. This *E*-pH was constructed with the assumption that the concentration of total dissolved copper ions [Cu^++^] was 10^−3^ mol·kg^−1^. The measurement results of the *E*_corr_ and pH solution in Figure 6 and Figure 7 are added in Figure 12. The stable chemical species is for a copper tube that was immersed in 10^3^ ppm Cu(COOH)_2_ solution with solution pH 5.5 (red line) are Cu_2_O and H_2_O. However, for the solution pH 3 (blue line), the stable chemical species are Cu^++^ and H_2_O at initial immersion; and after 20 days immersion, the stable chemical species are Cu(HCOO)_2_ and H_2_O. This is also the reason why copper tube that had been immersed in the 10^3^ ppm Cu(COOH)_2_ solution with solution pH 3 has a more prominent influence on the surface. 

In Section 4.2, Section 4.3 and Section 4.4, the detailed explanation of corrosion rate and corrosion mechanism are conducted for the condition of copper tube immersed for 20 days in 10^3^ ppm Cu(COOH)_2_ solution with pH 5.5. This condition is focused on because: (1) ANC occurred in 20 days immersion; therefore, it is important to explain clearly the corrosion rate in this condition. However, the corrosion rate in other conditions is mention in Table 2 as the summary of the electrochemical measurement values; (2) it was only Cu(COOH)_2_ solution without adding HCOOH. The complex ANC result in the experiment was in this condition.

### 4.2. The Comproportionation and Disproportionation Reaction in the Corrosion Mechanism

In previous research [27], the effect of copper formate on the ANC pitting attack on copper tube was reported. The corrosion mechanism of the copper tube in this condition is approximately close. The corrosion product was the same, and the comproportionation reaction, and disproportionation reaction occured in this condition. This paper is focused on the copper tube immersed in 10^3^ ppm Cu(HCOO)_2_ solution. Cu(HCOO)_2_ will be dissociated as follow:

Cu(HCOO)_2_ ⇆ Cu^2+^ + 2HCOO^−^ (1)

The dissociation constant (p*K*_a_) of HCOO^−^ is 3.55, Therefore, HCOO^−^ in Reaction (2) stays in the solution as a chemical form of HCOOH. In other words, the environment in the test solution is composed of mixtures of Cu^2+^, HCOOH, Cu(HCOO)_2_ and HCHO. The Cannizzaro reaction involved in the ANC in the HCOOH solution is a disproportionation reaction, which is expressed as follows:

2HCOOH ⟶ HCHO + H_2_CO_3_ (2)

The *E*-pH diagram in Figure 12 shows that the stable chemical species are Cu_2_O and H_2_O. Therefore, the copper tube immersed in this solution will change to Cu_2_O. The literature [8,16,26] reported that the ANC starts from the weak point of copper tube that does not have oxide film. However, the sample treatment is explained in Section 2.1 and means a new oxide film after the treatment be produced. Therefore, the oxide film such as CuO and Cu(OH)_2_ on the copper tube surface will also be changed into the Cu_2_O by the following comproportionation reaction of Equation (6) via the acid-base reactions of Equations (3)–(5):

CuO + H_2_O ⟶ Cu^2+^ + 2OH^−^ (3)

Cu(OH)_2_ ⟶ Cu^2+^ + 2OH^−^ (4)

Cu^2+^ +2HCOO^−^ ⟶ Cu(HCOO)_2_ (5)

Cu + Cu(HCOO)_2_ + H_2_O ⟶ Cu_2_O + 2HCOOH (6)

The DO as a reduction reaction is also involved at the early stage.

O_2_ + 2H_2_O + 4e^−^ ⟶ 4OH^−^ (7)

The consuming electron in the oxidation reaction by Equation (7) is considered as a pair reaction of the oxidation reaction of HCHO, which is shown as below:

HCHO + H_2_O ⟶ HCOOH +2H^+^ +2e^−^ (8)

Summation shows the total reaction in Equation (7) Moreover, (8):

2HCHO + O_2_ ⟶ 2HCOOH (9)

Therefore, the DO in the process of the ANC will be consumed by the oxidation of HCHO, especially in the pitting hole. Reaction (7) as a cathode reaction is hardly involved in the whole process for the ANC, except for the early immersion time. Sakai et al [30] reports that the dissolved oxygen is one of the strong influences in the mechanism of the copper tube. The DO will probably not always become a major cathodic reaction for the ANC process not only because of the consumption of DO by Equation (9) but it will also be difficult to access the narrow pitting hole with complex hole routes. As shown in Figure 4 and Figure 5, the result of EPMA analysis indicates that the oxygen in the pitting hole in 30 days was less than oxygen in the pitting hole in 15 days. The following comproportionation reaction had an important role in the deep pitting attack, which is expressed as Equation (6).

Dividing of the Reaction (6) into the redox reactions:

(10) $$2\mathrm{Cu}\;+{\;H}_{2}O\;⟶{\;\mathrm{Cu}}_{2}O\;+\;2H^{+}\;+\;2e^{-}$$

(11) $$2\mathrm{Cu}{\left(\mathrm{HCOO}\right)}_{2}\;+{\;H}_{2}O\;+\;2e^{-}\;⟶{\;\mathrm{Cu}}_{2}O\;+\;\mathrm{HCOOH}\;+\;2{\mathrm{HCOO}}^{-}$$

Cu_2_O as the reaction product is considered to act as a highly positive catalyst for the Cannizzaro reaction in the pitting hole. The coexistence of HCHO, H_2_CO_3_, and HCOOH in the narrow pitting hole probably is as major a factor for unstable corrosion environment as a change in the solution pH. 

### 4.3. Polarization Curve and Polarization Resistance Curve Analysis

The experimental values are obtained by reading Figure 8, Figure 9, Figure 10 and Figure 11 shown in Table 2. The values of *E*(0), *h*(0), αz_a_, βz_b_, *i*_red,L_, *i*_Ox,L_ and *i*_a0_ of the copper tube in both conditions are mentioned in this table. Figure 10 and Figure 11 show that there are no straight line with a gradient of $\frac{d\;\log h}{d\;\log i}=-1$. Therefore, it is classified as a fast system [31]. Seri et al [32] report observations about the clasification of irreversible, reversible and quasi reversible reaction. Since all the polarization resistance curve (Figure 10 and Figure 11) have a longer horizontal line with smaller *h*(0), this can be classified as a resersible reaction which means that the value of αz_a_ and βz_b_ approximated 1. The values of *i*_Ox,L_ are from the polarization resistance curve (Figure 10 and Figure 11). The value of *i*_a0_ at 20 days after immersion in both condition is higher than at initial immersion (0 day).

As mentioned in Section 4.1, an obvious explanation is needed in the condition of copper tube immersed for 20 days in 10^3^ ppm Cu(COOH)_2_ solution with pH 5.5, especially in terms of the calculation about the theoretical value of the measurement. Due to the characteristics of *E*(*i*), copper tube immersed for 20 days in 10^3^ ppm Cu(COOH)_2_ solution with pH 5.5 is classified as a fast system. The fast system then leads us to the spontaneous situation described as the following spontaneous Equations [31]:

(12) $$i=i_{a}+i_{c}$$

(13) $$h\left(i\right)=\frac{1}{1/h_{a}\left(i_{a}\right)+1/h_{c}\left(i_{c}\right)}$$

(14) $$h_{a}(i_{a})=\frac{RT}{zF}\frac{1}{i_{a}}+r_{A}=\frac{0.013}{i_{a}}+r_{A}$$

(15) $$h_{c}\left(i_{c}\right)=\frac{RT}{zF}\left(\frac{2}{-i_{c}}+\frac{1}{i_{c}-i_{\mathrm{Cu}{\left(\mathrm{HCOO}\right)}_{2},L}}\right)+r_{C}$$

(16) $$i_{a}\left(i\right)=i_{a}\left(0\right)+\frac{h\left(0\right)}{h_{a}\left(i_{a}\left(0\right)\right)}\cdot i$$

(17) $$i_{c}\left(i\right)=i_{c}\left(0\right)+\frac{h\left(0\right)}{h_{c}\left(i_{c}\left(0\right)\right)}\cdot i$$

where *r*_A_ and *r*_C_ represent the physical resistances such as an oxide film. In this experiment, we could assume that the oxide film resistance of the anode direction and cathode reaction are almost the same; $r_{A}=\;r_{C}$. The ANC is categorized as the fast system. Therefore, the $i_{\mathrm{Cu}{\left(\mathrm{HCOO}\right)}_{2},L}$ as the anodic branch rate in Equation (11) can participate in the corrosion rate as the limiting current density, because the activity of the metallic copper shows a unit and there is plenty of water as reactant. So, the total net current *i* is simplified as:

(18) $$i=i_{a}+i_{c}=i_{a}+i_{\mathrm{Cu}{\left(\mathrm{HCOO}\right)}_{2},L}$$

In this situation, *h*(*i*) will be arranged into:

(19) $$h\left(i\right)=\frac{dE}{di}=\frac{dE}{d\left(i_{a}+i_{\mathrm{Cu}{\left(\mathrm{HCOO}\right)}_{2},L}\right)}\approx h_{a}\left(i_{a}\right)$$

Rearranging Equation (13), moreover (14), we can obtain the following relation:

(20) $$h\left(i\right)=h_{a}\left(i-i_{\mathrm{Cu}{\left(\mathrm{HCOO}\right)}_{2},L}\right)=\frac{0.013}{i-i_{\mathrm{Cu}{\left(\mathrm{HCOO}\right)}_{2},L}}+r_{A}$$

The theoretical value for the $i_{\mathrm{Cu}{\left(\mathrm{HCOO}\right)}_{2},L}$ in 10^3^ ppm $\mathrm{Cu}{\left(\mathrm{HCOO}\right)}_{2}$ at the stagnant state (δ≈0.05 cm) can be calculated as:

(21) $$\begin{array}{cc} i_{\mathrm{Cu}{\left(\mathrm{HCOO}\right)}_{2},L} & =-zFk_{\mathrm{Cu}{\left(\mathrm{HCOO}\right)}_{2}}{\left[\mathrm{Cu}{\left(\mathrm{HCOO}\right)}_{2}\right]}_{\mathrm{bulk}}\\ & =-zF\frac{D_{\mathrm{Cu}{\left(\mathrm{HCOO}\right)}_{2}}}{\delta }{\left[\mathrm{Cu}{\left(\mathrm{HCOO}\right)}_{2}\right]}_{\mathrm{bulk}}\\ & \approx -\left(1\right)\left(96.5\times 10^{3}{\;A\;s\;\mathrm{mol}}^{-1}\right)\times \left(\frac{10^{-5}{\;\mathrm{cm}}^{2}s^{-1}}{0.05\;\mathrm{cm}}\right)\left(\frac{10^{3}{\;g\;\mathrm{dm}}^{-3}}{153{\;g\;\mathrm{mol}}^{-1}}\right)\\ & \approx -0.12{\;\mathrm{mA}\;\mathrm{cm}}^{-2} \end{array}$$

In this case, Equation (20) is corrected to:

(22) $$h\left(i\right)=\frac{0.013}{i+0.12}+r_{A}$$

Using the above equation, the theoretical *h*(0) value is calculated as:

(23) $$h\left(0\right)=\frac{0.013}{0.12}+r_{A}=0.11{\;k\Omega \;\mathrm{cm}}^{2}+r_{A}$$

On the other hand, the experimental *h*(0) value shown in Figure 10 is 2.7 kΩ cm^2^, and then *r*_A_ will be estimated by *r*_A_ = *h*(0) − 0.072 = 2.7 − 0.11 ≈ 2.6 kΩ cm^2^. Then Equation (22) is expressed as:

(24) $$h\left(i\right)=\frac{0.013}{i+0.12}+2.6$$

The equation above tells us that the ANC in the steady state will be influenced by the diffusion limiting current of $\mathrm{Cu}{\left(\mathrm{HCOO}\right)}_{2}$, $i_{\mathrm{Cu}{\left(\mathrm{HCOO}\right)}_{2},L}\approx -0.12{\;\mathrm{mA}\;\mathrm{cm}}^{-2}$, and oxide film resistance of *r*_A_ = 2.6 kΩ cm^2^.

### 4.4. The Relationship between h(i) and E(i) Curve

The characteristics of *E*(*i*) of the copper tube in 10^3^ ppm Cu(COOH)_2_ solution with pH 5.5 mean it is categorized as a fast system. In addition, if the *h*(*i*) in Equation (24) is correct, the experimental *E*(*i*) curve in Figure 8 will agree with the curve when theoretically solving the differential equation of *h*(*i*) at an initial condition of (0 mA cm^−2^, *E*(0)). Since the *E*_ocp_ in the steady state (20 days immersion) indicates around 0.04 V, the equation is concretely shown as:

(25) $$\int_{E\left(0\right)}^{E\left(i\right)}dE=\int_{0}^{i}\left(\frac{0.013}{i+0.18}+2.6\right)di$$

To solve the above Equation (25), the following relation is obtained:

(26) $$E\left(i\right)\;=E\left(0\right)+0.013\;\ln\frac{i+0.12}{0.12}+2.6i=0.04+0.013\;\ln\frac{i+0.12}{0.12}+2.6i$$

**Figure 13:**
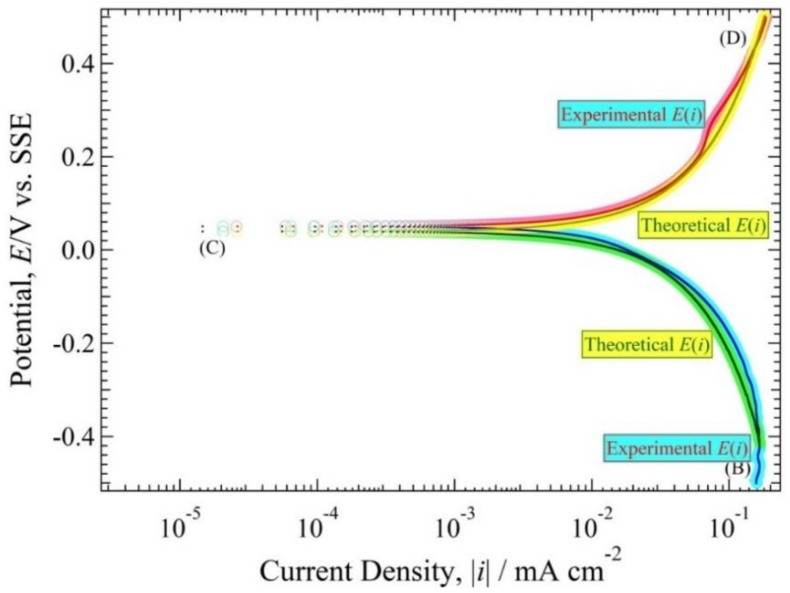
Superimposition of the experimental polarization curve and theoretical polarization curve obtained by solving the polarization resistance equation.

The comparison figure of the theoretical curve by Equation (26) to the experimental curve in Figure 8 is shown in Figure 13. The theoretical curve overlapped with the experimental curve indicates that theoretical analysis using the polarization resistance technique is almost correct and is useful for describing the behavior in this experiment. 

## 5. Conclusions

Ant nest corrosion on copper tube in 10^3^ ppm Cu(HCOO)_2_ solution occurred after 20 days both in solutions pH 5.5 and 3. Decreasing pH solution shrinks the amount of ANC on the copper tube. The stable chemical species of copper tube in 10^3^ ppm Cu(HCOO)_2_ solution with solution pH 5.5 were Cu_2_O and H_2_O. The comproportionation and disproportionation reaction critically influence the mechanism of ant nest corrosion. In the late immersion time, the concentration of oxygen was less. Therefore, it was difficult for the oxygen to enter the pitting hole and the comproportionation reaction plays a big role in this condition and makes it occur continuously. 

The polarization curve of the copper tube specimen was measured at an initial immersion (0 days) and late immersion time (20 days). It was shown that the polarization curve for 0 days and 20 days do not appear changed. Analysis using the polarization resistance curves revealed that the corrosion rate of the copper tube in Cu(HCOO)_2_ solution is extremely fast. In addition, the diffusion limiting current of $\mathrm{Cu}{\left(\mathrm{HCOO}\right)}_{2}$, $i_{\mathrm{Cu}{\left(\mathrm{HCOO}\right)}_{2},L}$ and oxide film resistance influenced the ant nest corrosion mechanism in the steady state.

## Figures and Tables

**Table 1:** Thermodynamic data (1 atm, 298 K).

Substance Formula	State	Chemical Potential, *μ^ϕ^*/kJ mol^−1^
HCHO	aq	−131
HCOOH	aq	−372
HCOO^−^	aq	−351
H_2_CO_3_	aq	−623
HCO_3_^−^	aq	−587
CO_2_	aq	−386
H_2_O	l	−237
OH^−^	aq	−157
Cu(OH)_2_	aq	−249
CuO	cr	−130
Cu_2_O	cr	−146
Cu^2+^	aq	65
Cu^+^	aq	50
Cu(HCOO)^+^	aq	−297
Cu(HCOO)_2_	aq	−636

**Table 2:** Experimental value obtained by reading Figure 8, Figure 9, Figure 10 and Figure 11.

Items	pH 5.5		pH 3 (Addition HCOOH)		Remarks
	0 Day	20 Days	0 Day	20 Days	20 Days
*E*(0)	0.04 V vs. SSE	0.04 vs. SSE	0.04 V vs. SSE	0.04 V vs. SSE	measured
*h*(0)	3.2 kΩ cm^2^	2.7 kΩ cm^2^	1.8 kΩ cm^2^	0.8 kΩ cm^2^	measured
αz_a_	1	1	1	1	approximated
βz_b_	1	1	1	1	approximated
*i*_red,L_	∞	∞	∞	∞	unknown
*i*_Ox,L_	−0.17	−0.15	−0.11	−0.2	approximated
*i*_a0_	8.1 × 10^−3^ mA cm^−2^	9.6 × 10^−3^ mA cm^−2^	1.4 × 10^−2^ mA cm^−2^	3.2 × 10^−2^ mA cm^−2^	corrosion rate

## Abbreviations

*i* is the net current density (*i* = *i*_a_ + *i*_a_) (mA cm^−2^)
*i*_a_ is the anodic branch current density (mA cm^−2^)
*i*_a_ is the cathodic branch current density (mA cm^−2^)
*h*_a_(*i*_a_) is the anodic branch polarization resistance (kΩ cm^2^)
*h*_c_(*i*_c_) is the cathodic branch polarization resistance (kΩ cm^2^)
*z* is the number of electrons transferred (-)
*F* is the Faraday’s constant (*F* = 96.5 × 10^3^ A s mol^−1^)
*R* is the gas constant (*R* = 8.31 J mol^−1^ K^−1^)
*T* is the absolute temperature (K)
δ is the Nernst diffusion layer thickness (cm)
$i_{\mathrm{Cu}{\left(\mathrm{HCOO}\right)}_{2}}$ is the limiting diffusion current density of $\mathrm{Cu}{\left(\mathrm{HCOO}\right)}_{2}$ (mA cm^−2^)
${\left[\mathrm{Cu}{\left(\mathrm{HCOO}\right)}_{2}\right]}_{\mathrm{bulk}}$ is the activity of $\mathrm{Cu}{\left(\mathrm{HCOO}\right)}_{2}$ in the bulk solution (-)
$k_{\mathrm{Cu}{\left(\mathrm{HCOO}\right)}_{2},L}$ is the limiting diffusion rate constant of $\mathrm{Cu}{\left(\mathrm{HCOO}\right)}_{2}$ (cm s^−1^)
$D_{\mathrm{Cu}{\left(\mathrm{HCOO}\right)}_{2}}$ is the limiting diffusion coefficient of the $\mathrm{Cu}{\left(\mathrm{HCOO}\right)}_{2}$ (cm^2^ s^−1^)
